# Early PSA Change after [^177^Lu]PSMA-617 Radioligand Therapy as a Predicator of Biochemical Response and Overall Survival

**DOI:** 10.3390/cancers14010149

**Published:** 2021-12-29

**Authors:** Felix Kind, Thomas F. Fassbender, Geoffroy Andrieux, Melanie Boerries, Philipp T. Meyer, Juri Ruf

**Affiliations:** 1 Department of Nuclear Medicine, Faculty of Medicine, Medical Center—University of Freiburg, University of Freiburg, Hugstetter Str. 55, D-79106 Freiburg, Germany; Thomas.Fassbender@ortenau-klinikum.de (T.F.F.); philipp.meyer@uniklinik-freiburg.de (P.T.M.); juri.ruf@uniklinik-freiburg.de (J.R.); 2 Institute of Medical Bioinformatics and Systems Medicine, Faculty of Medicine, Medical Center—University of Freiburg, University of Freiburg, Breisacher Str. 153, D-79110 Freiburg, Germany; geoffroy.andrieux@uniklinik-freiburg.de (G.A.); melanie.boerries@uniklinik-freiburg.de (M.B.); 3 German Cancer Consortium (DKTK) Partner Site Freiburg, German Cancer Research Center (DKFZ), Im Neuenheimer Feld 280, D-69120 Heidelberg, Germany

**Keywords:** castration refractory metastatic prostate cancer, PSMA, PSA, radioligand therapy, [^177^Lu]PSMA-617

## Abstract

**Simple Summary:**

Radioligand therapy with [^177^Lu]PSMA-617 (PSMA-RLT) is a promising therapeutic option for metastatic castration-resistant prostate cancers (mCRPC), as its clinical relevance has recently been confirmed in the phase III VISION-trial. As prostate-specific antigen (PSA) plays an important role in the response evaluation of this therapy, and the aim of this study was to prospectively assess the prognostic value of early PSA measurements. We found PSA changes as early as four weeks after the first administration of PSMA-RLT to be predictive of both long-term biochemical and PET imaging response, as well as overall survival. We then evaluated relevant predictive thresholds in PSA change at that time point, as the early detection of long-term (non-)response to PSMA-RLT can be of great benefit in the clinical management of terminally ill mCRPC-patients.

**Abstract:**

Purpose: Radioligand therapy with [^177^Lu]PSMA-617 (PSMA-RLT) is a promising therapeutic option for metastatic castration-resistant prostate cancer (mCPRP). This study assessed the prognostic value of early PSA measurements during PSMA-RLT. Methods: 27 patients with mCRPC scheduled for PSMA-RLT were prospectively enrolled for a serial short-interval PSA-assessment. Change in PSA (∆%PSA) during two treatment cycles was correlated with biochemical response (BR) and change in tumor volume on PET (TV) after 16 weeks (w16), as well as overall survival (OS). PCWG3 criteria and the recently recommended threshold of ∆%PSA ≤ −30% were assessed for their predictive value. Results: ∆%PSA first correlated with BR, TV and OS after 4 weeks (c1w4). At c1w4, ∆%PSA ≤ −30% was associated with the biochemical response at w16 (*p* = 0.003) and a longer median OS (*p* = 0.025), whereas the PCWG3-derived threshold of ∆%PSA ≤ −50% showed no such correlation. In contrast, ∆%PSA ≥ 25% at c1w4 was associated with biochemical progression at w16 (*p* = 0.003) and a shorter median OS (*p* < 0.001). Conclusion: PSA changes as early as four weeks after PSMA-RLT allow a significant prediction of later biochemical and PET-based imaging response, as well as OS. At this early time point, a more lenient threshold for a PSA decrease of at least 30% appears better-suited for the prediction of a positive biochemical response and longer OS. In contrast, the PCWG3-derived threshold for PSA increase (+25%) reliably anticipates biochemical progression and shorter OS.

## 1. Introduction

Prostate cancer comes in second place in both incidence and cause of all cancer-related deaths for men worldwide [1]. For advanced metastatic castration-resistant prostate cancer (mCRPC), in particular, survival rates are still low [1] as therapeutic options are limited. Radionuclide therapy targeting the prostate-membrane specific antigen (PSMA)—a class II membrane glycoprotein frequently overexpressed by prostate cancer cells [2]—with ^177^Lu-labelled radioligands (PSMA radioligand therapy—PSMA-RLT) is a promising treatment [3,4,5], and its efficacy in the mCRPC-setting has recently been confirmed in the phase III VISION-trial [6].

Beside image-based assessment, the prostate-specific antigen (PSA) plays an important role as a biochemical marker in the evaluation of therapeutic response and clinical follow-up of prostate cancer [7]. In PSMA-RLT, the role of imaging with, for instance, CT or bone scans can be limited [8], as—in the case of diffuse or disseminated metastatic disease—the full extent of the disease may not be reflected, and bone scans may not change in appearance early on, despite progression or response. Additionally, recently suggested criteria for PSMA PET/CT-based response evaluation have yet to be validated [9]. In clinical trials, as well as clinical routine, changes in PSA are, therefore, frequently used to evaluate response to PSMA-RLT [3,4,10]. The PSA response is generally categorized in accordance with criteria recommended by the Prostate Cancer Working Group (PCWG).

While current PCWG recommendations (PCWG3) suggest PSA evaluation at twelve weeks after treatment at the earliest [7] (mostly based on the evaluation of chemotherapy with taxanes [11,12]), more recent studies on PSMA-RLT [10,13,14] and second-generation androgen receptor inhibitors (ARI) [15] indicate that biochemical differentiation of therapy responders from non-responders might be possible after shorter time intervals.

Despite a reported plasma half-life of PSA of two to three days [16], we incidentally observed some major PSA changes in individual patients treated with PSMA-RLT as early as only a few days after the first cycle of treatment. While random PSA variations are known in a physiological or low tumor burden setting over longer periods of time [17], these distinct short-term changes were surprising. We, therefore, conducted a study assessing the prognostic value of early PSA measurements in patients undergoing PMSA-RLT. The aim of this study was to determine the earliest time point at which PSA change becomes predictive of long-term biochemical response as well as survival.

## 2. Materials and Methods

### 2.1. Patient Population

Between June 2017 and January 2019, 27 patients (median age 73 ± 8.3 years; range 54–86 years) with mCRPC were enrolled for prospective short-interval PSA assessment during PSMA-RLT (German Clinical Trial Register DRKS00013500), which was approved by the local institutional review board (Vote no.: 504/16). All patients gave written informed consent. Eligibility criteria used for PSMA-RLT were in accordance with the recommendations by current guidelines on RLT-usage [18,19]. Non-mCRPC status, prior therapy with PSMA-RLT or the inability to adhere to the time schedule of PSA determination (e.g., logistical reasons in case of patients from abroad) were exclusion criteria for study participation.

### 2.2. Treatment Regime and PSA Measurement

**Figure 1:**
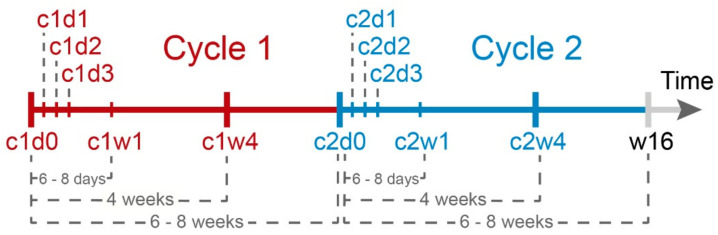
Treatment schedule of PSMA radioligand therapy and study design (designation of time and intervals of PSA measurements). c: therapy cycle, w: week of therapy cycle and d: day of therapy cycle.

Radiosynthesis of the radioligand PSMA-617 was performed as previously described [20], with slight modifications, including the use of n.c.a. [^177^Lu]LuCl3, ammonium acetate buffer and 67-nmol DOTA-PSMA-617 (ABX advanced biochemical compounds, D-01454 Radeberg, Germany). Two cycles of PSMA-RLT (at 6 GBq (162.16 mCi) per cycle) were administered six to eight weeks apart, followed by biochemical (PSA) and PSMA PET/CT-based restaging six to eight weeks later (i.e., 16 weeks after first administration). Subsequently, following in-house standards, patients who showed no obvious signs of progression (clinically or on PET/CT) or high-grade toxicity received further cycles of RLT, while patients with clinical and/or imaging-based progression were offered the best standard of care treatment. To assure consistency and comparability, prospective PSA assessment in this study was therefore confined to the first two treatment cycles up to restaging. For each cycle (c1, c2), serum PSA (Total PSA: Elecsys^®^ total PSA, Roche Diagnostics GmbH, D-79639 Grenzach-Wyhlen, Germany) was determined at our facility during the 3- to 4-day hospitalization period immediately before administration (d0), as well as 24 (d1), 48 (d2) and 72 (d3) hours later; during ambulatory visits the following week (i.e., 4–7 days after administration, w1); during follow-up examination four weeks after administration (w4) and at the restaging after two cycles (w16). See Figure 1 for the study timeline and naming conventions.

The change of PSA at each time point relative to the baseline (c1d0) was calculated using the following formula:

$$\Delta {\%\mathrm{PSA}}_{timepoint}=\frac{{\mathrm{PSA}}_{timepoint}-{\;\mathrm{PSA}}_{baseline}}{{\mathrm{PSA}}_{baseline}}*100\;\left[\%\right]$$

Whole-body PSMA PET scans were acquired before therapy and at w16. Additionally, to assess the clinical treatment effects, the pain status was assessed at c1d0 and w16, utilizing the “Quality of Life Questionnaire C30” (QLQ-C30) developed by the “European Organization for Research and Treatment of Cancer” (EORTC). Pain intensity was estimated on a verbal rating scale of “none”, “a little”, “quite a bit” and “very much” [21].

### 2.3. Efficacy and Response/Endpoints

Referring to PCWG3 recommendations, PSMA-RLT was considered a cytotoxic treatment. Therefore, the primary control/relieve/eliminate endpoint was the biochemical response (BR) at w16, with a quantified change of the whole-body tumor volume at w16 as a secondary endpoint for validation. Additionally, overall survival (OS) was assessed for all enrolled patients in September 2021 (51 months after the inclusion of the first patient). Following the PCWG3 criteria [7], BR at w16 was categorized as either progression (∆%PSA ≥ 25%), stable (−50% < ∆%PSA < 25%) or response (∆%PSA ≤ −50%). In accordance with the PCWG3 guidelines, the PSA endpoint response was confirmed by an additional follow-up assessment (≥3 weeks later). In addition to the aforementioned PCWG3 standards and in keeping with more recent studies, especially [10,22,23], PSA measurements during the first two therapy cycles were also categorized by an a priori defined response criterion of PSA decrease of at least 30% (∆%PSA ≤ −30%) to better account for potentially still-evolving therapeutic effects at early time intervals. Whole-body tumor volume at the baseline and w16 was assessed by a semiautomated analysis of PSMA PET/CT acquisitions using Fiji [24], an ImageJ2-based [25] image processing package, and the Beth Isreal plugin [26]. Volumes of interest (VOI) were defined via the auto-segmentation function. In accordance to Seifert et al. [27], a volumetric assessment of each VOI was done by percental thresholding (50% of maximal SUV). The resulting volumes of all relevant lesions were then summed to determine the whole-body PSMA tumor volume (TV50). Change in the tumor volume (∆%TV50) was calculated by the following formula:

$$\Delta \%\mathrm{TV}50=\frac{\mathrm{TV}50_{w16}-\mathrm{TV}50_{baseline}}{\mathrm{TV}50_{baseline}}*100\;\left[\%\right]$$

### 2.4. Statistical Analysis

SPSS 24.0.0.0 was used for statistical analyses. Data were presented as the mean ± standard deviation, range or 95% confidence intervals (95% CI). Normal distribution was assessed using Shapiro–Wilk tests. Mixed analysis of variance (ANOVA) was used to analyze and compare serial PSA change during the first two cycles between patient groups categorized by BR at w16, employing Greenhouse–Geisser adjustment to correct the violations of sphericity and Levene’s test to assess the homogeneity of error variances, as well as Welch’s *t*-tests and Tukey’s HSD post-hoc analysis when appropriate. Chi-square test, utilizing Cramer-V, and Fisher’s exact test were used to assess the cross-table correlation between the qualitative PSA responses, categorized by different thresholds, after four and 16 weeks. Univariate linear regression was applied to correlate the PSA change at given time points with the change in tumor volume at w16, utilizing adjusted R^2^ (adj. R^2^) for the effect size assessment. The relation between various baseline parameters and the primary endpoints was evaluated using linear regression for changes in the PSA at w16 and Cox regression models for the OS, with only significantly univariate variables (*p* ≤ 0.05) included in the multivariate analysis. Kaplan–Meier analysis and Cox proportional hazard regression were applied to determine associations between changes in the PSA and OS, utilizing log-rank tests and hazard ratios (HR) to compare survival data between the biochemical response groups. Finally, an exploratory retrospective receiver operating characteristic (ROC) analysis, including Youden’s J, was used to retrospectively ascertain the early PSA thresholds, predictive of BR at w16.

## 3. Results

In 27 patients, 59 cycles of PSMA-RLT were administered with an average dose of 5.99 ± 0.12 GBq (5.59–6.20, 151.08–167.57). Detailed patient characteristics, including previous therapies, are given in Supplemental Table S1. All patients received at least one cycle of PSMA-RLT. Four out of 27 patients (15%) were excluded from further analysis, as PSMA-RLT was discontinued by patients’ request after one cycle, the reason being clinical progression at a follow-up examination (c1w4) in three patients and refusal of further treatment (despite non-progression) in one patient. Of the remaining 23 study patients, 16 (59%), 5 (19%) and 2 (7%) patients received two, three and four cycles, respectively.

At w16, 16 patients (59%) achieved a biochemical response according to the PCWG3 criteria (mean ∆%PSA_w16_ −77 ± 13). Four patients (15%) displayed PSA progression (mean ∆%PSA_w16_ 103 ± 62), and three patients were biochemically stable (mean ∆%PSA_w16_ −19 ± 22). In all patients, the biochemical response status was confirmed in the PSA follow-up. ∆%PSA_w16_ had no correlation to previous treatments or other relevant pretherapeutic parameters (*p* ≥ 0.15; Supplemental Table S2). Patient follow-up was of over 51 months, with the median follow-up (reverse Kaplan–Meier estimator) being 39.1 months (95% CI 32.1–46.1). Median survival was 12.0 (95% CI 10.8–13.2) months, with five patients (19%) still alive at the last follow-up. There were no therapy-related deaths documented. At w16, 14 patients (52%) stated an improvement in pain status from the baseline of at least one tier/level, 11 of which were biochemical responders. Simultaneously, only two patients, one biochemically progressive and the other stable, reported a rise in pain levels. Correspondingly, concerning baseline pain medication, a switch from nonsteroidal anti-inflammatory drugs (NSAID) to opioids at w16 was only necessary in three patients (stable, *n* = 1; progressive, *n* = 2), while, concurrently, former opioid intake could be reduced to NSAID in five responders.

According to a multivariate Cox regression model based on prior univariate Cox regression analyses of relevant pretherapeutic parameters, only PSA (log10 transformed, *p* = 0.012) and TV50 (*p* = 0.014) at the baseline were predictive of OS (negative correlation). Detailed univariate and multivariate cox regression results are shown in Table 1.

Additionally, independent univariate Cox regression showed that the number of PSMA-RLT cycles correlated positively with the OS (HR 0.55 (95% CI 0.38–0.81), *p* = 0.002).

### 3.1. Early PSA Change with Regard to Biochemical Response

#### 3.1.1. Early PSA Change and Biochemical Response after 16 Weeks

**Figure 2:**
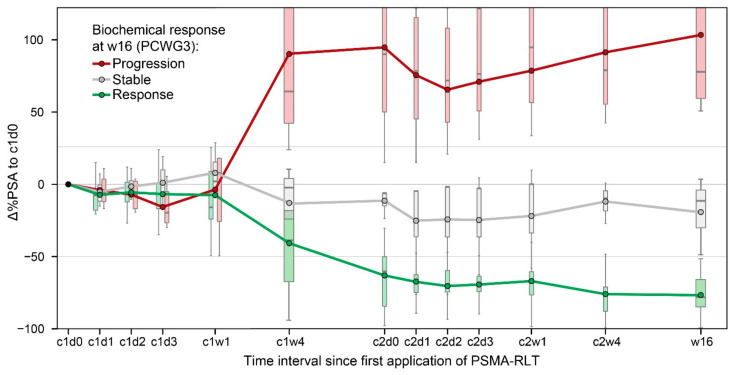
Percental change in PSA (Δ%PSA) one day (c1d1), two days (c1d2), three days (c1d3), one week (c1w4) and at restaging 16 weeks (w16) after first administration (c1d0) of ^177^Lu-labelled radioligand therapy (PSMA-RLT), categorized by the biochemical response at w16 following PCWG3 criteria. Response: PSA decrease of at least 50%, Stable: PSA decrease smaller than 50% or PSA increase smaller than 25% and Progression: PSA increase of at least 25%. Mean values (open circles), and box plots are given for each time point.

A mixed ANOVA was performed, differentiating the PSA development of the later biochemical response (according to PCWG3) over eleven time points across two cycles. Please also refer to Figure 2 for details.

Due to the expected dependence between within-subjects variables (∆%PSA) and between-subjects factors (response), there was a statistically significant interaction between the time and response groups (F(4.61, 46.09) = 17.39, *p* < 0.001, partial η^2^ = 0.64). ∆%PSA differed significantly between the response groups, from four weeks after the first administration of PSMA-RLT (c1w4) until the end of the observation period (*p* < 0.001). At c1w4, each response group was distinguishable by ∆%PSA from the other two groups (Tukey HSD *p* < 0.015).

Earlier changes in the PSA during the first two weeks after administration showed no such significant correlation with long-term BR, as PSA values were fluctuant (mean ∆%PSA_c1d1–c1w1_ −5 ± 18, range −49 to 69) but approximately normally distributed (*p* > 0.095).

#### 3.1.2. Biochemical Response Categorization by Early PSA Change

**Figure 3:**
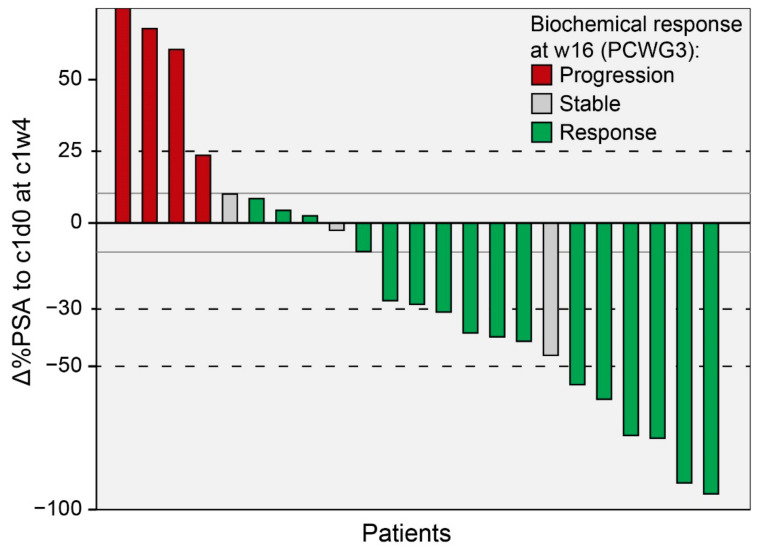
Waterfall plot of percental PSA change (Δ%PSA) four weeks (c1w4) after the first administration of PSMA-RLT (relative to the baseline), with bar coloring according to the biochemical response at restaging after 16 weeks (w16) categorized by PCWG3 criteria. Response: PSA decrease ≥ 50%, Stable: PSA decrease ≤ 50% or PSA increase ≤ 25% and Progression: PSA increase ≥ 25%. Dashed lines represent a priori defined thresholds for responses classified at c1w4. Grey, nonbroken lines represent thresholds with the highest discriminatory power at c1w4 (±10%), calculated by a retrospective ROC analysis.

At c1w4, PSA thresholds of ∆%PSAc1w4 ≥ 25% and ∆%PSA_c1w4_ ≤ −30% were significant and strong predictors for biochemical progression (χ2(4) = 16.39 *p* < 0.001, *n* = 23; Cramer-V = 0.844) and the response at w16 (χ^2^(4) = 4.54, *p* = 0.033, *n* = 23; Cramer-V = 0.44), respectively. At that time point, ∆%PSA_c1w4_ ≥ 25% showed a sensitivity of 75% (3/4) and specificity of 100% (19/19) for biochemical progression, while ∆%PSA_c1w4_ ≤ −30% offered a sensitivity of 63% (10/16) and a specificity of 86% (6/7) for the biochemical response. The respective contingency table and waterfall plot comparing the two time points (c1w4 and w16) are given in Table 2 and Figure 3, respectively.

Data are presented as the number of patients with percentage of patients per row in parentheses. ^a^ PSA decrease of at least 50%, ^b^ PSA decrease smaller than 50% or PSA increase smaller than 25% and ^c^ PSA increase of at least 25%; χ^2^(4) = 11.91, *p* = 0.003, Cramer-V = 0.622.

In contrast to these two thresholds, the PCWG3-derived response criterion of ∆%PSA_c1w4_ ≤ −50% narrowly failed to be a significant predictor of the future biochemical response (*p* = 0.059), as only six out of the 16 responders at w16 were correctly categorized (sensitivity 38%, specificity 100%; Supplemental Table S3).

#### 3.1.3. Exploratory Retrospective ROC Analysis

The ROC analysis of ∆%PSA_c1w4_ (dependent variable) revealed ∆%PSA_c1w4_ ≤ −10% to have the highest discriminatory power for the biochemical response at w16, providing a sensitivity of 81% (13/16) and specificity of 87% (6/7) (area under curve (AUC) 0.88 (95% CI 0.68–0.98), *p* < 0.001, Youden’s J 0.67). Conversely, a ∆%PSA_c1w4_ > 10% proved optimal for the detection of biochemical progression at w16, with a sensitivity and specificity of 100%, respectively (4/4, 19/19) (AUC 1.0 (95% CI 0.85–1.0), *p* < 0.001, Youden’s J 1.00).

### 3.2. Early PSA Change with Regard to Change in Tumour Volume

∆%PSA at w16 correlated strongly with ∆%TV50 (F(1,21) = 59.69, *p* < 0.001, adj. R^2^ = 0.73). A corresponding significant correlation with ∆%TV50 for early ∆%PSA occurred for the first time at c1w4 (F(1,21) = 8.85, *p* = 0.007, adj. R^2^ = 0.26).

### 3.3. Early PSA Change with Regard to Overall Survival

To assess the relation between PSA change during the first two cycles of PSMA-RLT and OS, continuous univariate Cox regression was used for all time points until restaging (w16). c1w4 was again the first time point at which ∆%PSA was significantly associated with OS (HR 1.007 (95% CI 1.001–1.013), *p* = 0.015).

**Figure 4:**
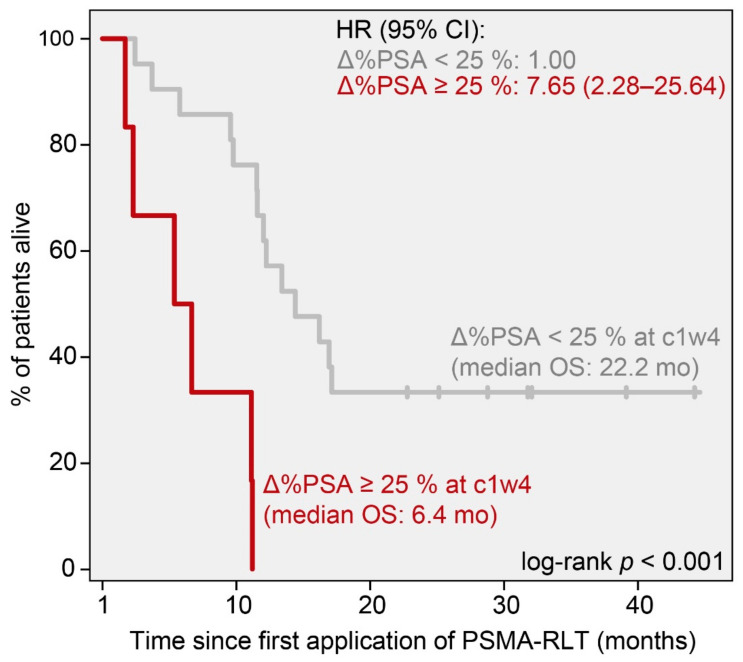
Kaplan–Meier curves for the overall survival (OS) of patients stratified by a PSA increase of at least 25% (Δ%PSA ≤ 25%) at four weeks (c1w4). Median OS and hazard ratios (HR) with 95% confidence interval (95% CI) are given for each group.

**Figure 5:**
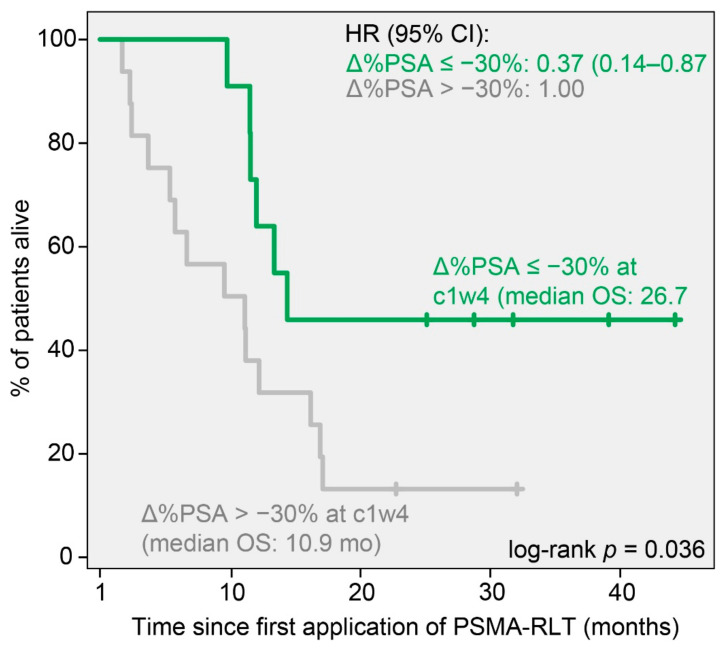
Kaplan–Meier curves for the OS of patients stratified by a PSA decrease of at least 30% (Δ%PSA ≤ −30%) at four weeks (c1w4). Median OS and hazard ratios (HR) with 95% confidence interval (95% CI) are given for each group.

Kaplan–Meier analyses at that time point revealed ∆%PSA_c1w4_ ≥ 25% to be significantly associated with shorter median OS (6.4 (95% CI 3.1–9.7) months vs. 21.8 (95% CI 14.6–28.9) months, *p* < 0.001; HR 7.63 (95% CI 2.28–25.64), *p* = 0.001; Figure 4), while patients with a PSA decrease of ∆%PSA_c1w4_ ≤ −30% exhibited significantly longer median OS (27.8 (95% CI 17.1–38.4) months vs. 12.0 (95% CI 6.9–17.1) months, *p* = 0.025; HR 0.37 (95% CI 0.15–0.91), *p* = 0.031; Figure 5) than all the other patients.

This differentiation of OS persisted up until w16 for both thresholds: ∆%PSA_w16_ ≥ 25% (6.2 (95% CI 3.0–9.2) months vs. 23.7 (95% CI 17.2–29.4) months *p* < 0.001; HR 7.7 (95% CI 0.14–0.97), *p* < 0.001) and ∆%PSA_w16_ ≤ −30% (24.6 (95% CI 16.4–32.7) months vs. 11.0 (95% CI 6.8–15.2) months, *p* = 0.038; HR 0.36 (95% CI 0.13–0.87), *p* = 0.045).

For the PCWG3-derived response criterion of ∆%PSA ≤ −50%, an association with a significantly longer median OS was only observed at w16 (25.8 (95% CI 17.8–33.9) months vs. 11.3 (95% CI 7.7–14.9) months, *p* = 0.039; HR 0.36 (95% CI 0.13–0.95), *p* = 0.048) and was not found at earlier time points, e.g., at c1w4 (*p* = 0.507).

Using the PSA thresholds identified by exploratory retrospective ROC analyses, patients with a PSA decrease of at least 10% at c1w4 (∆%PSA_c1w4_ ≤ −10%) exhibited significantly longer median OS (26.3 (95% CI 16.5–36.0) months) than all the other patients (∆%PSA_c1w4_ > −10%; 10.9 (95% CI 5.9–15.9) months, *p* = 0.024; HR 0.39 (95% CI 0.16–0.91), *p* = 0.03). Conversely, a PSA increase of more than 10% (∆%PSA_c1w4_ > 10%) was significantly associated with a shorter median OS (7.4 (95% CI 3.7–11.1) months vs. 22.9 (95% CI 15.3–30.6) months, *p* = 0.002; HR 4.1, (95% CI 1.6–10.2), *p* = 0.003).

## 4. Discussion

The aim of the present study was to assess the prognostic value of early changes in PSA after PSMA-RLT based on incidental observations of significant fluctuation days after clinical administration.

Concordant with recent prospective data [3,5], the biochemical response rate to PSMA-RLT was high and showed no significant correlations to either individual previous treatments or the number of preceding systemic therapy lines or to the pretherapeutic biochemical and clinical parameters, respectively. There was a relevant improvement in theclinical status, i.e., pain status, and the need for pain medication decreased over the course of therapy—in particular, among biochemical responders. Multivariate Cox regression confirmed the role of PSA as an independent predictor of OS. Additionally, the volumetric tumor burden (TV50) was another baseline predictor of OS, and its change after two therapy cycles (∆%TV50) correlated strongly with the corresponding change in PSA (∆%PSA). Taken together, these observations suggest that PSA is a sufficiently reliable and easily determinable surrogate parameter for the early prediction of response to PSMA-RLT, despite the fact that neither PSA decline nor tumor shrinkage are formally hard, FDA-relevant parameters for drug approval [7].

A systematic analysis of ∆%PSA up to week two after the first administration showed variable changes fluctuating around zero, with no significant correlation to the primary endpoints (BR at w16 and OS) or the secondary endpoint (∆%TV50 at w16). Thus, no prognostic information can be derived from ∆%PSA assessments during this early period.

However, beginning in week four after the first treatment, continued periodic ∆%PSA assessments revealed a significant correlation between the PSA change and BR and OS, respectively. At that time point, ∆%PSA thresholds of −30% and 25% were highly predictive of both BR at w16, as defined by the PCWG3 criteria [7], as well as OS.

A PSA increase of at least 25% after four weeks was especially prognostic of both a shorter OS (3.5 times) and biochemical progression at w16 (specificity 100%), as PSA flare-up (PSA increase of at least 25% succeeded by PSA decrease at w16), occurring, e.g., in antihormonal treatment [28], was not observed at that time point. The early detection of a long-term nonresponse to PSMA-RLT may have clinical implications for the treatment evaluation of mCRPC patients. As quality of life plays an important role in oncology, a switch to the best supportive care has to be considered as an alternative to a potentially taxing and time-consuming therapy if there is little prospect of a clinical response [29].

A PSA decrease threshold of −30% after four weeks was predictive for the biochemical response and a significantly longer median OS (27.8 vs. 12.0 months). This more lenient, a priori defined threshold for PSA decrease was evaluated in accordance with data from other recent studies [10,22,23]. In contrast, a threshold for PSA decreases of −50% after 4 weeks, more in-line with PCWG3 recommendations for a biochemical response assessment after at least 12 weeks [7], was not significantly associated with the primary endpoints. A possible reason for this observation could lie in not yet fully consolidated therapeutic effects at that early point in treatment. This observation is strengthened by coinciding data from retrospective studies with larger sample sizes, which report the suitability of this lower threshold for the early response assessment of PSMA-RLT [10,30,31]. Interestingly, these results also coincide with findings of early PSA assessment during second-generation ARI treatment, which recommend the very same 30% decrease threshold at the same time point, namely 4 weeks after therapy initiation [15].

Corresponding to that idea, we also retrospectively performed exploratory ROC analyses to ascertain PSA changes at week four, with the optimal discriminatory power for BR at week 16. In-line with our previous observations, these analyses provided even lower PSA thresholds, as PSA changes of more than ±10% were most indicative of both the future biochemical response and progression, respectively, while also being significantly associated with the corresponding differences in the OS. However, considering the high variability of the PSA levels we observed in the first weeks after the first treatment cycle, the reliability of such low thresholds has to be assessed very carefully and needs to be validated in future prospective studies.

This study had some limitations. Despite the prospective nature of our serial short-interval PSA assessment, PSMA-RLT itself was still performed under clinical routine conditions. Moreover, besides the relatively small sample size, the results may be biased by the heterogeneity of previous and, especially, further therapies. For instance, treatment was primarily continued in responsive patients, demonstrated, for example, by the significant correlation between the OS and the number of administered cycles. In addition, despite the concordance of the biochemical and PET-based quantified imaging responses, an established assessment system integrating, e.g., PCWG criteria and PET is still warranted. Moreover, due to logistical reasons (i.e., long distance to hospital), the PSA at week three of each cycle could not be evaluated systematically.

## 5. Conclusions

This study shows that PSA changes in patients with mCRPC as early as four weeks after the first administration of PSMA-RLT are predictive of both long-term biochemical and PET imaging responses, as well as overall survival. At this early stage, a PSA decrease of at least 30% was found to be more suitable to detect future responders, as well as a longer overall survival compared to the threshold of 50% proposed by the PCWG for long-term biochemical evaluation. An early increase in PSA (+25%) is a strong predictor for biochemical progression and shorter overall survival. Larger prospective studies are warranted to validate these findings and provide criteria for the early assessment of PSMA-RLT, including even more lenient thresholds, as suggested by our explorative retrospective ROC analysis.

## Figures and Tables

**Table 1:** Cox regression model of overall survival with various pretherapeutic variables (see Supplemental Table S1).

	Univariate Analysis		Multivariate Analysis	
	*p*	HR (95% CI)	*p*	HR (95% CI)
UICC Stage at diagnosis	0.423			
Postsurgical Gleason Score	0.854			
Previous Treatments				
Radical Prostatectomy	0.586			
Radiotherapy	0.55			
Androgen Deprivation	0.41			
Abiraterone	0.571			
Enzalutamide	0.63			
[^223^Ra]Radiumdichloride	0.111			
Docetaxel	0.564			
Cabazitaxel	**0.04**	7.22 (1.7–30.54)	0.893	
number of previous systemic therapy lines *	0.904			
Age	0.723			
Lymph Node Metastases	0.471			
Performance Status (Karnofsky index)	**0.012**	0.91 (0.85–0.98)	0.156	
HB (g/dL)	**0.002**	0.74 (0.61–0.90)	0.249	
LDH (U/L)	**0.002**	1.004 (1.001–1.007)	0.428	
PSA (ng/mL)	**0.034**	1.76 (1.34–2.47)	**0.012**	1.78 (1.03–3.09)
Tumor volume on PET	**0.01**	1.004 (1.002–1.006)	**0.014**	1.003 (1.001–1.006)

Bold numbers denote statistical significance. If not stated otherwise, a parameter is ascertained at the baseline of PSMA RLT; * binary categorization (“more than three lines of systemic treatment”); Abbreviations (alphabetically): HB: hemoglobin concentration, LDH: lactate dehydrogenase, UICC: Union Internationale Contre le Cancer.

**Table 2:** Correlation between early PSA change (Δ%PSA) four weeks after first administration of PSMA-RLT (baseline) and biochemical response at restaging after 2 cycles using a threshold of early PSA decrease of 30% to baseline (Δ%PSA ≤ −30%).

Δ%PSA 4 Weeks after First Administration	Biochemical Response at Restaging According to PCWG3 Criteria			
	Response ^a^	Stable ^b^	Progression ^c^	Total
Δ%PSA ≤ −30%	10 (91%)	1 (9%)	-	11
−30% < Δ%PSA < 25%	6 (67%)	2 (22%)	1 (11%)	9
Δ%PSA ≥ 25%	-	-	3 (100%)	3
Total	16	3	4	*n* = 23

## Data Availability

Raw data were generated at the Department of Nuclear Medicine, Medical Center—University of Freiburg, Germany. The data presented in this study are available upon reasonable request from the corresponding author. The data are not publicly available due to privacy restrictions.

## Supplementary Materials

The following are available online at https://www.mdpi.com/article/10.3390/cancers14010149/s1: Supplemental Table S1: Baseline patients’ characteristics, *n* = 27. Supplemental Table S2: Results of univariate linear regressions of various pretherapeutic variables with changes in the PSA during restaging after two cycles of [^177^Lu]PSMA-617 radioligand therapy. Supplemental Table S3: Correlation between the early changes in PSA four weeks after baseline (first administration of PSMA-RLT) and the biochemical response during restaging after two cycles of [^177^Lu]PSMA-617 radioligand therapy, using a threshold of early PSA decrease of 50% compared to the baseline (Δ%PSA ≤ −50%).
