# Supplementary material for: Early PSA Change after [177Lu]PSMA-617 Radioligand Therapy as a Predicator of Biochemical Response and Overall Survival
Source: Cancers (Basel). 2021 Dec 29;14(1):149. doi: 10.3390/cancers14010149 (PMC8750166; doi:10.3390/cancers14010149)
Supplement: Supplementary file 1 [file cancers-14-00149-s001.zip › Supplemental Table S1.pdf]

Supplemental Table S1: Baseline patients' characteristics, n=27

Patient no.	Age at diagnosis [years]	UICC stage at diagnosis	Postsurgical Gleason score	Previous Treatments (Number of previous systemic therapy lines)	Age at baseline [years]	Known Metastases / Infiltration at baseline	Performance status (Karnofsky index)	Analgesic intake at baseline	HB at c1d0 [g/dl]	LDH at c1d0 [U/l]	PSA at c1d0 [ng/ml]	TV50 at baseline [ml]
**1**	60	4	8	RAD, ADT, DOC (2)	62	OSS	90		13.8	260	10.0	7.1
**2**	54	4	9	RPE, RAD, ADT, ABI, ENZA, DOC (4)	62	LYM, OSS, ADR, REC	90	OP	10.5	282	639.8	98.3
**3**	70	2	9	RPE, RAD, ADT, ABI (2)	77	LYM, OSS	100		11.1	394	98.3	39.3
**4**	62	3	9	RPE, RAD, ADT, ABI, ENZA, DOC, Ra^{223}	70	LYM, OSS	100		12.5	193	384.0	51
**5**	68	3	9	RAD, ADT, ENZA (2)	73	LYM, OSS	100	NSAID	12.0	317	86.0	45.2
**6**	60	4	7	RPE, RAD, ADT, ABI, ENZA, DOC,	81	LYM, OSS	90	OP	10.1	450	1708.0	87.1
**7**	75	4	8	ADT, DOC (2)	80	LYM, OSS, PUL	90	NSAID	6.1	503	2749.0	565.9
**8**	66	1	7	RAD, ADT, ABI, ENZA, DOC (4)	75	LYM, OSS, PUL, HEP	90	OP	7.8	476	61.2	156.7
**9**	71	4	9	RAD; ADT, ENZA, DOC, Ra^{223} (4)	77	LYM, OSS, PUL, BRA	100	NSAID	12.3	175	704.4	144.1
**10**	51	4	9	RPE, RAD, ADT (1)	54	LYM, OSS, PUL	90	OP	9.1	301	626.5	220.2
**11**	48	4	7	RPE, ADT, ABI, DOC (3)	65	OSS	100	OP	13.7	126	224.0	40.1
**12**	72	2	9	RPE, RAD, ADT (1)	84	LYM, OSS,	90		12.6	188	229.2	18.7
**13**	69	4	9	RAD, ADT, ENZA (2)	73	LYM, OSS, OTH	70	OP	11.0	204	101.4	312.7
**14**	52	4	7	RAD, ADT, ABI, ENZA, DOC, CABA (5)	56	LYM, OSS	90	OP	8.8	1499	176.8	432.4
**15**	62	4	8	RAD, ADT, ENZA, DOC (3)	83	LYM, OSS, PUL	90		14.6	180	7.6	16.8
**16**	60	4	9	RPE, RAD (0)	63	LYM, OSS	100		13.0	171	3.2	34.6
**17**	73	3	9	RPE, RAD, ADT, ABI, DOC (3)	83	LYM, OSS, REC, VES	90		10.2	259	71.4	23.3
**18**	67	4	9	RPE, RAD, DOC (1)	70	LYM, OSS	100		10.5	198	23.5	29.1
**19**	57	3	7	RPE, RAD, ADT (1)	75	LYM, OSS, PUL	90		13.4	218	28.1	47.7
**20**	63	4	9	RPE, ADT, ABI (2)	76	LYM, OSS, PUL, HEP	90		12.4	217	69.2	37.9
**21**	66	4	8	ADT, ABI, ENZA, DOC (4)	71	OSS	100		9.7	493	3168.0	88.5
**22**	57	3	8	RPE, RAD, ADT, ABI, DOC, CABA (4)	65	LYM, OSS, PUL	100	OP	9.6	649	770.1	711.9
**23**	60	3	9	RPE, RAD, ADT, DOC (2)	65	OSS	90	NSAID	10.5	298	95.2	73.4
**24**	66	3	8	RPE, RAD, ADT, ABI, ENZA, DOC (4)	76	LYM, OSS	90		13.6	173	103.0	42.8
**25**	62	3	9	RPE, RAD, ADT, ABI, ENZA, DOC, Ra^{223}	71	LYM, OSS	100		12.5	193	17.98	5.6
**26**	67	4	9	ADT, ABI, ENZA, Ra^{223} (4)	75	OSS	90	NSAID	11.4	168	597.6	167.1
**27**	73	4	7	RPE, ADT (1)	86	LYM, OSS, VES	100	OP	12.7	210	33.7	15.9

Abbreviations (alphabetically): ABI: Abiraterone, ADR: Metastasis of adrenal gland, ADT: Androgen deprivation therapy, BRA: Cerebral metastases, CAB: Complete androgen blockade, CABA: Cabazitaxel, DOC: Docetaxel, ENZA: Enzalutamide, HB: hemoglobin concentration, HEP: Hepatic metastases, LDH: Lactate dehydrogenase, LYM: Lymph node metastases, NSAID: nonsteroidal anti-inflammatory drugs, OP: Opiods, OSS: Bone metastases, OTH: Other locations, PSA: Prostate-specific antigen, PUL: Pulmonal metastases, Ra^{223}: [223Ra]Radiumdichloride, RAD: Radiotherapy, REC: Rectal infiltration, RPE: Radical prostatectomy, UICC: Union internationale contre le cancer, VES: Infiltration of bladder
