# Supplementary material for: Early PSA Change after [177Lu]PSMA-617 Radioligand Therapy as a Predicator of Biochemical Response and Overall Survival
Source: Cancers (Basel). 2021 Dec 29;14(1):149. doi: 10.3390/cancers14010149 (PMC8750166; doi:10.3390/cancers14010149)
Supplement: Supplementary file 1 [file cancers-14-00149-s001.zip › Supplemental Table S2.pdf]

Supplemental Table S2: Results of univariate linear regressions of various pre-therapeutic variables with change in PSA at restaging after two cycles of [^{177}Lu]PSMA-617 radioligand therapy

	**p**
**UICC Stage at diagnosis**	0.45
**Postsurgical Gleason Score**	0.763
**Previous Treatments**	
**Radical Prostatectomy**	0.333
**Radiotherapy**	0.867
**Androgen Deprivation**	0.656
**Abiraterone**	0.471
**Enzalutamide**	0.185
**[^{223}Ra]Radiumdichloride**	0.15
**Docetaxel**	0.719
**Cabazitaxel**	0.603
**number of previous systemic therapy lines ***	0.278
**Age**	0.195
**Lymph Node Metastases**	0.235
**Performance Status (Karnofsky index)**	0.649
**HB [g/dl]**	0.721
**LDH [U/l]**	0.969
**Tumor volume on PET**	0.61

If not stated otherwise, parameter is ascertained at baseline of PSMA RLT; *binary categorization ("more than three lines of systemic therapy"); Abbreviations (alphabetically): HB: hemoglobin concentration, LDH: Lactate dehydrogenase, UICC: Union internationale contre le cancer
