# Supplementary material for: Early PSA Change after [177Lu]PSMA-617 Radioligand Therapy as a Predicator of Biochemical Response and Overall Survival
Source: Cancers (Basel). 2021 Dec 29;14(1):149. doi: 10.3390/cancers14010149 (PMC8750166; doi:10.3390/cancers14010149)
Supplement: Supplementary file 1 [file cancers-14-00149-s001.zip › Supplemental Table S3.pdf]

Supplemental Table S3: Correlation between early changes in PSA (Δ%PSA) four weeks after first administration of PSMA-RLT (baseline) and biochemical response at restaging after two cycles of [177Lu]PSMA-617 radioligand therapy, using a threshold of early PSA decrease of 50% to baseline (Δ%PSA ≤ -50%).

**Δ%PSA 4 weeks after first administration**	**Biochemical response at restaging according to PCWG3 criteria**		
	**Response[a]**	**Non-Response[b]**	**Total**
**Δ%PSA ≤ -50%**	6 (100%)	-	6
**Δ%PSA > -50%**	10 (59%)	7 (41%)	17
**Total**	16	7	n= 23

*data are presented as number of patients with percentage of patients per row in parentheses

[a] PSA decrease of at least 50%, [b] PSA decrease less than 50 % or PSA increase; Fisher's exact test: p = 0.059
